# A narrative review of tuberculosis in the United States among persons aged 65 years and older

**DOI:** 10.1016/j.jctube.2022.100321

**Published:** 2022-06-13

**Authors:** Iris L. Wu, Amit S. Chitnis, Devan Jaganath

**Affiliations:** a School of Public Health, University of California, Berkeley, Berkeley, CA, United States; b School of Medicine, Virginia Commonwealth University, Richmond, VA, United States; c Tuberculosis Section, Division of Communicable Disease Control and Prevention, Alameda County Public Health Department, San Leandro, CA, United States; d Division of Pediatric Infectious Diseases, University of California, San Francisco, San Francisco, CA, United States; e Center for Tuberculosis, University of California, San Francisco, CA, United States

**Keywords:** Epidemiology, Prevention, Diagnosis, Drug therapy, Tuberculosis, Aged

## Abstract

Tuberculosis (TB) is a preventable infectious disease that confers significant morbidity, mortality, and psychosocial challenges. As TB incidence in the United States (U.S.) decreased from 9.7/100,000 to 2.2/100,000 from 1993 to 2020, the proportion of cases occurring among adults aged 65 and older increased. We conducted a review of published literature in the U.S. and other similar low-TB-burden settings to characterize the epidemiology and unique diagnostic challenges of TB in older adults. This narrative review also provides an overview of treatment characteristics, outcomes, and research gaps in this patient population. Older adults had a 30% higher likelihood of delayed TB diagnosis, with contributing factors such as acid-fast bacilli sputum smear-negative disease (56%) and non-classical clinical presentation. At least 90% of TB cases among older adults resulted from reactivation of latent TB infection (LTBI), but guidance around when to screen and treat LTBI in these patients is lacking. In addition, routine TB testing methods such as interferon-gamma release assays were two times more likely to have false-negative results among older adults. Advanced age was also often accompanied by complex comorbidities and impaired drug metabolism, increasing the risk of treatment failure (23%) and death (19%). A greater understanding of the unique factors of TB among older adults will inform clinical and public health efforts to improve outcomes in this complex patient population and TB control in the U.S.

## 1 Introduction

TB remains an important preventable infectious disease that poses significant clinical and public health challenges in the United States (U.S.). In northern California, active TB disease has been associated with 7% mortality within one year of diagnosis and 16.3% mortality beyond one year, with an average of seven years of potential life lost [1]. In 2018, approximately 5,000 TB-related hospitalizations occurred in the U.S. [2] with an average length of stay of nine days and costs ranging from $10,100-$45,400 [3], [4].

TB incidence in the U.S. has declined annually since 1992, with incidence decreasing from 9.7/100,000 to 2.2/100,000 from 1993 to 2020 [5], [6]. Several important demographic and clinical shifts in TB cases have occurred during this period. TB cases increasingly occur among non-U.S.-born individuals and racial and ethnic minorities [6], are more likely to be attributed to reactivation of latent TB infection (LTBI) rather than recent exposure, and frequently arise among clinically complex individuals [7]. Many of these trends are closely related to the increasing median age of TB disease. Adults aged 65 years and older comprise an increasing proportion of TB cases, representing more than a quarter of all cases in the U.S. since 2017 [5]. This patient population presents unique challenges for TB prevention and treatment. Older patients are often frailer at baseline and have significant comorbidities that increase the risk for TB treatment failure and death. Many older individuals also reside in congregate settings, increasing the potential risk of TB transmission and challenges for infection control.

More TB cases are occurring among older and clinically complex patients, but few papers detail epidemiology and outcomes in this population. This review seeks to identify and characterize patterns in the epidemiology, diagnosis, treatment, and outcomes of both TB and LTBI in the U.S. for adults aged 65 years and older (hereafter referred to as older adults or older individuals). This information will guide strategies for diagnosis, treatment, and prevention of TB for this population and identify future opportunities for further research and intervention.

## 2 Methods

A literature search was conducted during October – December 2021 within the PubMed database with search terms including “tuberculosis”, “older”, “United States”, “epidemiology”, “diagnosis”, “treatment”, and “dosing”. All relevant articles and their associated citations were reviewed. Studies from the U.S. were emphasized in this review, although relevant articles from similar low TB incidence countries were also included. There is a paucity of published literature focusing on this patient population, particularly around novel anti-TB agents, structural barriers to treatment, and post-TB health. A narrative review methodology and format was adopted to convey the current knowledge in the field and highlight future research needs. Additional incidence and epidemiologic data were obtained from U.S. Centers for Disease Control and Prevention (CDC) Online Tuberculosis Information System [8].

## 3 Epidemiology of TB in older adults

**Fig. 1:**
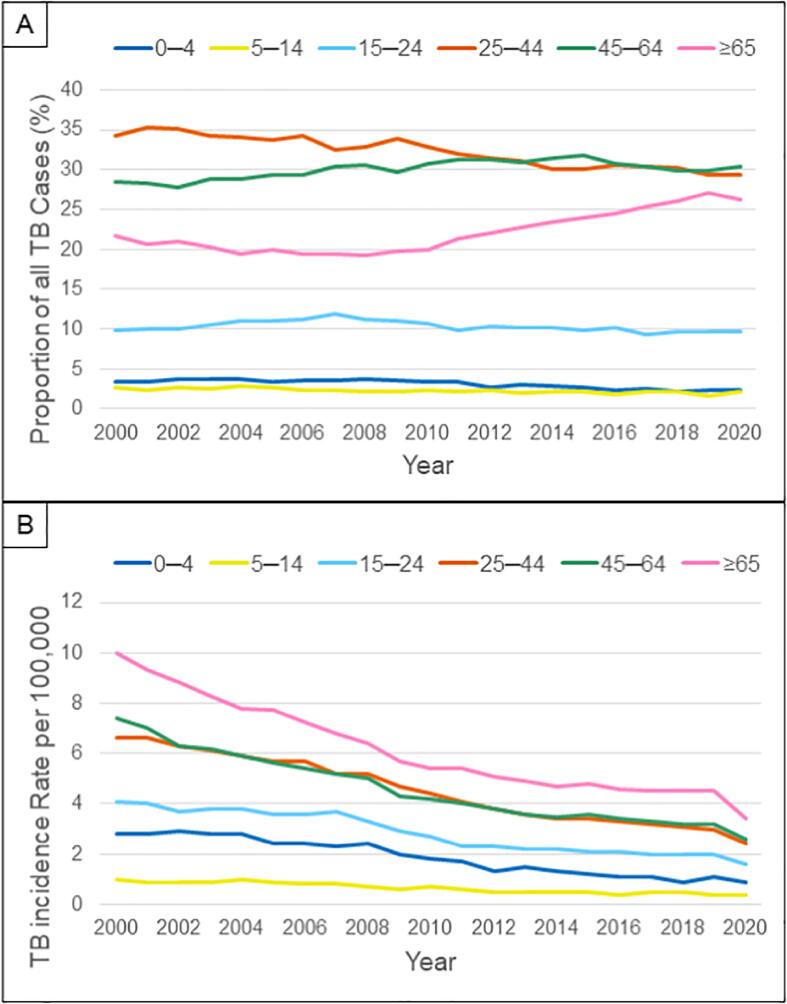
**Epidemiologic Trends in Tuberculosis by Age Group, United States, 2000**–2020.* [A] U.S. TB cases from 2000 to 2020 were stratified by age group to show an increasing proportion of cases among adults ≥ 65. [B] TB incidence rates declined between 2000 and 2020. However, rates among adults ≥ 65 are consistently higher than all other age groups. *Data queried from CDC Online Tuberculosis Information System.

While TB incidence has decreased overall in the U.S., incidence in adults aged 65 years and older has declined at a slower rate than other age groups [6]. The proportion of TB cases among older adults have consequently increased in the past decade (Fig. 1). Since 2017, older adults constituted more than 25% of all tuberculosis cases in the U.S. while only representing 16% of the population. The risk of TB disease among older adults also rose by 20% from 2010 to 2017 [9]. Several factors may explain this shift, including, in part, a cohort effect – among U.S.-born cases, there is a slower decline in rates among older birth cohorts [10]. Of note, CDC reported a 20% reduction in overall TB cases from 2019 to 2020, potentially due to decreased transmission and missed diagnoses from lock-down, social distancing, and travel restriction measures taken during the coronavirus disease pandemic [6]. However, many of the demographic characteristics of TB cases remained consistent from 2019 to 2020 for older adults.

Over 90% of TB cases among older individuals result from reactivation of LTBI rather than recent transmission [11]. Older patients are also more likely to have complicating factors such as extrapulmonary disease or multidrug-resistant disease [7]. Comorbidities common in older individuals such as immunosuppression, diabetes, hepatic impairment, and renal insufficiency are associated with increased disease severity, adverse medication events, and mortality [12], [13], [14], [15]. Several population-wide racial/ethnic disparities in TB are also reflected among the older adult subgroup. In 2020, 71% of TB cases across all age groups occurred among non-U.S.-born individuals [6]. Similarly, 75% of cases among older adults were reported to be non-U.S.-born individuals [8]. Incidence rates are highest among non-U.S.-born Asian and Pacific Islander older adults, consistent with all younger age groups [16].

Certain demographic trends among U.S.-born or institutionalized individuals differ within age groups. In 2020, U.S.-born cases aged 65 years and older were most likely to be non-Hispanic White, whereas younger U.S.-born cases were more likely to be Black or Hispanic [8]. Older patients were also more likely to reside in long-term care facilities (LTCF), although cases were vastly more prevalent in the community, with only 4.5% of all cases attributed to LTCF residents among this age group in 2019 [8].

Patients with TB have become increasingly older, non-U.S.-born, and clinically complex. Demographic shifts in LTBI and TB disease burden nationwide should change our approach to prevention, detection, and treatment. A more thorough understanding of characteristics and risk factors (summarized in Table 1) in this evolving patient population will allow clinicians and public health officials to improve disease prevention and screening.

**Table 1:** Epidemiologic, Demographic, and Clinical Characteristics of Patients Aged 65 Years and Older with Tuberculosis, United States.

**Type**	**Characteristics**
Epidemiologic	Older adults have represented over 25% of all TB* cases since 2017 [6], [15]. Over 90% of TB cases in older adults are from latent TB reactivation [10]. Diabetes and other risk factors for LTBI^†^ progression and severe TB disease are more common in older adults [11], [12], [13], [14]
Demographic	75% of TB cases in older adults occur in non-U.S.-born individuals [15]. TB incidence rates are highest in Asian and Pacific Islander populations [15]
Clinical	<30% of older adult patients with TB present with cough [17]. Older adults are more likely to present with non-specific, non-classic symptoms as weakness, weight loss, anorexia, dyspnea, and mental changes [18]

* Tuberculosis.

† Latent tuberculosis infection.

## 4 Diagnosing LTBI and TB disease in older adults

### 4.1 Clinical presentation

Older adults are more likely to present with non-specific clinical symptoms. One study noted only 28% of TB patients aged 65 years and older had cough, compared to 38% of younger adults (*p* = 0.035) [17]. This same study reported that older patients were less likely to have other classic symptoms such as fever (13% vs. 31% in younger adults, *p* = 0.001), night sweats (9% vs. 31% in younger adults, *p* = 0.001), or hemoptysis (2% vs. 5% in younger adults, *p* = 0.063). General, non-specific symptoms such as weakness (50% vs. 25% in younger adults, *p* < 0.001), weight loss (36% vs. 26% in younger adults, *p* = 0.045), anorexia (31% vs. 19% in younger adults, *p* < 0.001), dyspnea (39% vs. 22% in younger adults, *p* = 0.002), and mental changes (13% vs. 0.5% in younger adults, *p* < 0.001) were more frequent in older patients [18]. Clinicians may not have a high suspicion of TB when evaluating patients with atypical symptoms, particularly in older adults who are also at risk for malignancy or other similarly presenting conditions.

### 4.2 Screening and testing

Screening and testing for TB infection are usually performed with either a tuberculin skin test (TST) or interferon-gamma release assay (IGRA); however, older age is associated with higher odds of false-negative results due to various factors including immunosuppression and anergy. One *meta*-analysis found that the odds ratio (OR) of IGRA false negativity was two times higher in older individuals [19], while another study reported an OR of 1.69 for TST false negativity [20]. A large observational cohort study comparing TSTs and two IGRAs (QuantiFERON and T-SPOT.*TB*) reported concordance patterns that suggested higher IGRA sensitivities compared to TST among adults 65 and older [21]. Current practice guidelines recommend the use of IGRAs over TSTs in older patients [22].

### 4.3 Radiologic features

Older adults are less likely to exhibit classic TB radiologic findings. Studies show only 10% of older patients have classic findings of upper lobe involvement [23], and cavitation is less prevalent in older patients [24]. Older patients are 50% more likely to have other atypical features such as pleural effusions or thickening [23] and are twice as likely to have lower lung field involvement compared to younger patients [25]. In older adults who are also at risk for malignancy, inflammatory, or autoimmune diseases, atypical radiologic findings may create further confusion and diagnostic delays.

### 4.4 Microbiologic Work-Up

Microbiologic testing among older adults pose barriers in both sensitivity and specimen collection. Approximately 56% of older individuals have acid-fast bacilli sputum smear-negative TB, 1.2 times the prevalence in younger adults [26]. Sputum collection may also pose difficulties among older patients who are too frail to expectorate, or who are simply unable to produce a sufficient sample. Alternative specimens for TB detection have been proposed in sputum-scarce patients including stool, urine, gastric aspirates, and bronchial washings [27], [28], [29], [30]. While promising, these methods have rarely been investigated among older patients. An exploration and comparison of the sensitivity and specificity of alternative diagnostic methods among older individuals will help clinicians balance efficacy and invasiveness.

### 4.5 Missed diagnostic opportunities

All aforementioned factors coalesce to create significant barriers to timely diagnosis and treatment initiation among older patients. A 2021 study estimated that older individuals were 26% more likely to have a healthcare visit in which a diagnostic opportunity was missed [31]. Diagnostic delays have been hypothesized to be responsible for the increasing proportion of advanced TB cases in the U.S. [32]. While research into this association has produced mixed results [33], older individuals are more likely to experience treatment delays that may negatively affect disease outcomes. Some delays are attributed to misdiagnosis, as age 65 years and older is associated with 30% greater odds of misdiagnosis for both pulmonary and extrapulmonary TB disease [34]. Studies suggest that increased prevalence of altered mental status and higher suspicion of malignancy may lead clinicians to consider other diagnoses before testing for TB [35]. The threshold for obtaining chest imaging and diagnostic tests should be lowered among older adults who are more likely to have atypical disease presentations.

### 4.6 Structural barriers to diagnosis

In addition to clinical and laboratory challenges, older adults may contend with significant socioeconomic and structural barriers. Screening for LTBI remains inconsistent even though LTBI reactivation accounts for more than 90% of TB disease cases in this population. A study in the Boston, Massachusetts region documented that only 68% of LTCF residents underwent LTBI testing [36]. Approximately one-fifth of tested residents were positive for LTBI, but only a quarter of these positive cases were subsequently initiated on LTBI treatment. In California, TB incidence rates among community-dwelling older adults were higher than nursing home residents, with authors hypothesizing that LTBI screening and treatment occurred even less frequently outside of care facilities [37]. Older adults are also more likely to have incomplete diagnostic documentation and screening for risk factors such as HIV [38]. Many clinicians may be deterred from screening for LTBI in older adults whom they deem too frail to tolerate treatment.

A better understanding of non-classical features among older adults will improve timely diagnosis of TB in this population. Further research will be vital to decreasing diagnostic barriers. Increased education around LTBI screening is needed to prevent progression to TB disease.

## 5 Anti-tuberculosis treatment in older adults

### 5.1 Drug metabolism and interactions

Declining renal and hepatic function with age may pose difficulties in anti-TB drug metabolism. Older individuals are also more likely to have comorbid chronic diseases including diabetes, renal disease, and both HIV and non-HIV immunosuppression [24]. Treatment safety, tolerability, and efficacy are salient concerns for these patients. Therapeutic efficacy is difficult to establish in individuals with these comorbidities, as research has shown significantly lower plasma concentrations of anti-TB drugs in patients with diabetes or HIV co-infection [39], [40]. Medication safety is another challenge because many anti-TB drugs are metabolized by the liver. Rifamycins are particularly potent hepatic enzyme inducers that interact with many medications [41]. One study of hospitalized TB patients found over 50% of study participants had potential drug-drug interactions between their prescribed anti-TB regimen and their other medications [42]. Some studies suggest that rifabutin, a rifamycin with less potent hepatic enzyme induction, can be used instead of rifampin (RIF) or rifapentine when serious drug interactions are a concern [41]. In patients with comorbidities and baseline frailty, clinicians face significant challenges selecting an efficacious but safe anti-TB regimen.

### 5.2 Adverse medication events

Adverse drug reactions are twice as prevalent among older patients [18]. Hepatoxicity is a primary concern in older adults, with research showing that older patients have 70% greater odds of experiencing TB-drug-associated hepatic events [43]. The most prevalent adverse events in older patients are liver injury, hypersensitivity, gastrointestinal upset, and musculoskeletal complaints [24]. Among first-line TB drugs, pyrazinamide (PZA) is the most common culprit for adverse events in this population. Consequently, clinicians may avoid PZA in older adults by initiating a non-standard drug regimen, potentially placing patients at higher risk for treatment failure [44]. A randomized trial in Japan showed that use of PZA-containing regimens in patients aged 80 and older was not associated with higher mortality [45]. In fact, patients on non-PZA regimens had significantly longer time to culture conversion. Another study found non-PZA regimens more than tripled the odds of death [46]. These findings are reflected in American Thoracic Society/CDC TB treatment guidelines, which suggest that non-traditional regimens or non-PZA regimens should only be used in individuals without high-risk disease characteristics [47]. Although valid concerns of PZA tolerability exist for older patients, clinicians must carefully weigh the benefits and risks of adding PZA versus a second-line medication to maximize regimen efficacy.

Several newer drugs and regimens are changing the landscape of TB treatment and raising additional questions for the care of older adults. A trial in 2021 demonstrated non-inferiority of a four-month regimen of rifapentine, moxifloxacin, isoniazid, and PZA compared to the standard six-month RIPE regimen (RIF, isoniazid (INH), PZA, and ethambutol) [48]. However, only 35% of trial participants were older than 35 years (median age 31 years, range 13–81), and no significant age-stratified analyses on efficacy or safety were reported. For multidrug- and extensively drug-resistant TB, newer agents such as bedaquiline, linezolid, delamanid, and pretomanid are being utilized in oral regimens [49]. Several seminal clinical trials on these novel anti-TB agents notably did not include any participants aged 65 years or older [50], [51], [52]. Limited data exist around safety of these new agents in this population. A recent study evaluating QTc interval prolongation with combination bedaquiline and delamanid showed a modest, no more than additive effect, but did not include any participants older than 56 years [53]. More research is needed around the efficacy and safety of new medications and regimens in older adults.

### 5.3 LTBI treatment

Adverse medication events also challenge treatment completion for LTBI. One study examining claims data from 2005 to 2016 found the odds of LTBI treatment non-completion was five times greater among older adults compared to their younger counterparts on 6- and 9-month daily isoniazid (INH) regimens [54]. However, older patients residing in nursing homes have been successfully treated for LTBI with INH in the past [55], suggesting that frail patients can still tolerate LTBI treatment with careful monitoring.

The emergence of rifamycin-based regimens for LTBI have provided options that are better tolerated than 6 or 9 months of daily INH. These include weekly INH and rifapentine for 3 months (3HP), daily RIF for 4 months (4R), and daily INH and RIF for 3 months (3HR) [56]. Several randomized trials showed that rifamycin-based regimens exhibit similar treatment efficacy as daily INH while reporting significantly higher treatment completion and decreased hepatotoxicity [57], [58]. While none of these trials excluded older participants, only around 20% of study participants were aged 50 years or older. Generalization of these trial findings to older adults may be limited as age-stratified analyses are not available. However, findings from the PREVENT Tuberculosis study revealed that 3HP was associated with higher risk of systemic drug reactions (3.5% vs 0.4% with 9H), with age greater than 35 years as an independent risk factor [59]. The most common systemic drug reactions were flu-like (63%) or cutaneous (17%) symptoms. Little guidance exists for clinicians to navigate the initiation of LTBI treatment when balancing the risk of disease progression with the potential for adverse events and drug-drug interactions in older adults.

### 5.4 Medication dosing

Current anti-TB medication dosing recommendations are weight-based. Given risks for adverse medication events and concerns of frailty and low body weight in older patients, official guidelines recommend monthly weight monitoring to assess treatment response and adjust dosing [47]. In patients who are greater than 10% below ideal body weight, clinicians may need to prolong treatment if there is delayed mycobacterial eradication or clinical evidence of poor treatment response. However, some studies have suggested that dosing adjustments for low body weight leads to low plasma drug levels and treatment inefficacy, particularly for rifamycin drugs [60], [61]. Clinicians must balance the risk of adverse effects with the danger of treatment failure. Ideally, anti-TB drug levels should be monitored with serum specimens collected 2 and 6 h after each dose when drug malabsorption, under-dosing, or clinically important drug-drug interactions are suspected [41], [47]. However, therapeutic drug monitoring is difficult and may be impractical to implement in most outpatient treatment settings.

## 6 TB Disease outcomes in older patients

### 6.1 Treatment extension

TB treatment extensions are more likely among older adults due to refractory disease and treatment interruptions. One study conducted in New York City found 5.6% of older patients experienced treatment extensions compared to 4.6% of younger patients, with most delays occurring in cases of extrapulmonary disease [62]. Treatment extensions were commonly attributed to worsening or severe disease, use of nonstandard treatment regimens, or comorbidities. Risk of treatment non-completion is also higher, with one study in Washington State reporting 76.6% of older adults completed treatment compared to 94.9% in younger patients [24]. This disparity was heightened in study participants aged 75 years and older, among whom only 70.2% completed therapy. Death during treatment was a significant contributor to treatment noncompletion as age increased, with 28.1% of participants 75 and older who died during therapy compared to 9.3% aged 65–74. Lower treatment completion rates in older adults can be attributed to factors such as refractory disease, worsening comorbidities, adverse medication reactions, and death.

### 6.2 Death

In the U.S., CDC reported an overall TB mortality rate of 0.2 deaths/100,000 population in 2019 [5]. TB mortality has consistently declined since 1992 [5], but the decline has been attenuated in adults aged 75 years and older [63]. One study found that 18.9% older patients died during TB treatment compared to 2.1% of younger patients [24]. Another study reported up to 72% of deaths among older adults on TB treatment were TB-related [46]. Comorbidities common among older patients may contribute to this trend, as diabetes has been shown to increase risk of death by 35% [14]. Socioeconomic factors relevant to older adults, including institutionalization, social marginalization, and malnutrition, are major determinants of death following completion of anti-tuberculosis treatment [64].

### 6.3 Relapse

A greater risk of relapse could be hypothesized among older individuals, as age-related comorbidities such as diabetes can lead to delayed mycobacterial clearance and treatment failure [65]. Yet, data around TB recurrence in older adults are mixed. A national study from 1993 to 2006 found no age-associated increase in TB relapse 12 months after treatment completion [66]. Another study in South Carolina from 1970 to 2002 reported an OR of 1.88 for TB relapse in older adults [67]. However, the South Carolina study reported a high prevalence of suboptimal, non-rifamycin-based regimens. Differences in TB treatment regimens and demographics may limit comparison between these two studies. More research is needed into the mechanisms and trends of TB relapse among older adults.

### 6.4 Health and socioeconomic sequelae

Even after curative TB therapy, patients are often left with chronic physical, mental, and social sequelae. Studies have shown that pulmonary impairment during and after TB treatment does not differ significantly, suggesting that lung injury persists after disease eradication [68], [69]. Many patients also experience income loss, stigmatization, and social isolation during and after treatment [70]. Unfortunately, research into the health and socioeconomic impacts of TB disease has not been conducted specifically in older adults. For this patient population that is at risk of functional decline and social isolation, more research into post-TB impairments is needed to optimize health and quality of life.

## 7 Future directions

Our review has identified gaps in research and clinical guidelines around TB disease and infection among older adults (Table 2).

**Table 2:** Research Needs for Improved Detection and Treatment of Tuberculosis among Patients Aged 65 Years and Older.

**Area**	**Research Needs**
Diagnostics	New and improved diagnostics for TB* detection, particularly those utilizing alternative microbiologic specimens that can be reliably and non-invasively collected in older adults. Tools to predict LTBI^†^ progression to help clinicians identify high risk patients for treatment
Treatment	Testing of newer drugs in older adults to improve the safety, tolerability, and effectiveness of anti-TB regimens
Structural	Characterization of the impact of social determinants on TB susceptibility, severity, and outcomes

* Tuberculosis.

† Latent tuberculosis infection.

**Fig. 2:**
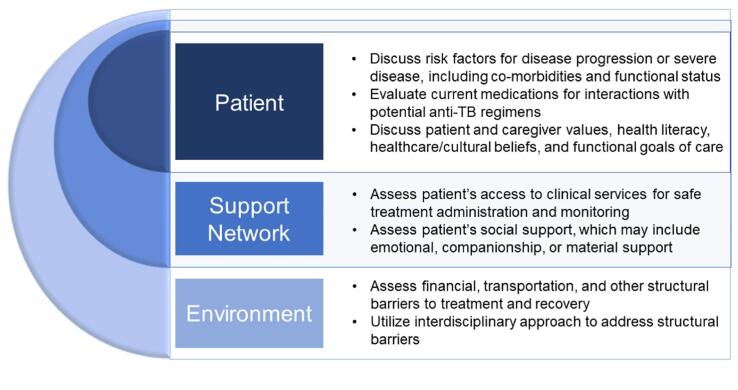
**Framework for Shared Decision-Making Around Screening and Treatment of Latent Tuberculosis Infection (LTBI).** Clinicians should engage in shared decision-making conversations with patients and caregivers around the subject of LTBI screening and treatment. Different individual, support network, and community factors that affect patient prognosis and outcomes should be discussed. Providers may need to schedule a longer visit with patients and caregivers to ensure adequate time for such nuanced conversations.

Given the challenges and complications of TB disease among older adults, prevention of TB reactivation is vital. However, several issues need to be addressed in future work. Current guidelines do not define an upper age limit for LTBI screening, and existing LTBI diagnostics often perform poorly in older adults. LTBI treatment initiation can be a difficult decision as we currently do not have reliable tools to predict which individuals will progress from LTBI to active TB disease. Patients have different health needs and functionality at every age, and shared decision-making around specific benefits and harms should be discussed by clinicians when screening and treating LTBI (Fig. 2). Clinicians and patients should also be aware that decisions not to treat LTBI will necessitate consideration of TB disease for future respiratory illnesses or other systemic concerns.

## 8 Conclusion

TB in the U.S. increasingly affects older adults. Absence of classic TB symptoms and indeterminate lab testing contribute to missed diagnoses and treatment delays in older patients. Medication selection and dosing pose challenges to clinicians balancing treatment efficacy with risk of adverse drug reactions. Older adults have higher risk of treatment failure and death. By highlighting these unique challenges in TB infection and disease in older adults, we hope to improve patient outcomes and TB prevention and control in the U.S.

## Declaration of Competing Interest

The authors declare that they have no known competing financial interests or personal relationships that could have appeared to influence the work reported in this paper.
